# Chronic Osteomyelitis of the tibia and ankle treated with Limb Salvage Reconstruction

**DOI:** 10.7150/jbji.40337

**Published:** 2019-12-10

**Authors:** Aaron Lam, Shawn S. Richardson, Josh Buksbaum, Jonathan Markowitz, Michael W. Henry, Andy O. Miller, S Robert Rozbruch, Austin T. Fragomen

**Affiliations:** 1 Maimonides Medical Center, 4802 10th Ave, Brooklyn, NY 11219, USA.; 2 Limb Lengthening and Complex Reconstruction Service, Hospital for Special Surgery, Weill Cornell Medical College, 535 East 70th Street, New York, NY 10021, USA.; 3 Emory University, 201 Dowman Dr, Atlanta, GA 30322, USA.; 4 Rush Medical College, 600 S. Paulina St., Chicago, IL 60612 USA.; 5 Infectious Disease Service, Hospital for Special Surgery, Weill Cornell Medical College, 535 East 70th Street, New York, NY 10021, USA.

**Keywords:** chronic osteomyelitis, limb salvage, limb preservation, amputation, tibia, ankle

## Abstract

**Introduction:** To confirm the success of our limb salvage treatment protocol and determine what factors are predictive of success versus failure in limb salvage techniques for patients with chronic osteomyelitis of the tibia and ankle.

**Methods:** Retrospective case series analyzing factors and outcomes in patients who underwent limb salvage techniques for chronic osteomyelitis of tibia or ankle. Main outcome measurements included infection controlled without the need for amputation or chronic antibiotic suppression and union of infected non-unions.

**Results:** Mean follow-up was 3.9 years. Out of the sixty-seven patients (mean age: 51.4 years) treated for chronic osteomyelitis, fifty-four had an associated non-union. Sixty-one patients (91.0%) had their infection controlled by limb salvage. Five ultimately required amputation and one remained on daily chronic antibiotics. Diabetics complicated with neuropathy and increasing numbers of limb salvage surgeries were associated with a significantly higher failure rate. Forty-eight out of fifty-four patients (88.9%) also had successful healing of their infected non-union. Diabetes and need for more limb salvage surgeries were also found to have a significantly higher failure rate.

**Conclusions:** Limb salvage is a reliable and successful treatment for patients with chronic osteomyelitis and infected non-unions of the lower extremities. Diabetic neuropathy is a risk factor that impedes success.

**Level of Evidence:** Prognostic Level IV.

## Introduction

Chronic osteomyelitis is one of the most challenging problems encountered by orthopaedic surgeons. In the setting of fracture or prosthetic joint replacement, infection typically stems from local contamination^13^. Open tibia and ankle fractures occur with an incidence of 3.4 per 100,000 and 1.6 per 100,000 respectively^5^,^7^, and have a high propensity to develop fracture related infection (FRI) with associated chronic osteomyelitis. Once infection is established, there is often associated fracture non-union, limb length discrepancy, and deformity. More often than not, chronic osteomyelitis cannot be cured without surgery^3^. Limb salvage remains the treatment goal for FRI with osteomyelitis. Various techniques have been reported in literature 1,2,11,12,15,18,19,22, but debate remains as to whether limb salvage techniques can be reliably more successful than amputation in providing patients with a painless, functional extremity with eradication of infection. Success rates for salvage have been documented between 62% to 100% with amputation rates at nearly 10%^8^,^10^,^17^. Amputation v. salvage guidelines available to patients and surgeons are limited. Further information is needed to document the success of limb salvage in order to assist with this decision.

The purpose of this study is to review the success of limb salvage in patients afflicted with chronic osteomyelitis and to determine what factors might predict outcome in these patients. The following questions were asked: (1) how successful was the treatment at controlling infection; (2) how successful was the treatment at healing infected non-unions; and (3) what were the predictors of failure of the treatment protocol in eradicating infection and healing non-unions?

## Methods

After obtaining institutional review board approval for a retrospective analysis, a combined database of the infectious disease (ID) and orthopedic services at a major academic center was queried to identify a consecutive series of 199 patients who had been treated between 2003 and 2018 by physicians from both services. A summary of the inclusion/exclusion criteria are listed in Table 1. Of the initial 199 patients, only 83 patients met the inclusion criteria. Another 16 were excluded because of missing records. The final number of patients enrolled in the study was 67.

Patients' medical charts and radiographs were reviewed. Demographic information including comorbidities and prior treatments were recorded. Key parameters collected included primary treatment method, number of limb salvage surgeries performed, use of local antibiotic (antibiotic cement-coated nails or beads), need for soft tissue coverage, and presence of non-union. Information on the individual infection and treatment protocol including cultured organism, total number of samples collected, number of positive samples, type and duration of acute intravenous (IV) antibiotics used, and type and duration of chronic daily oral (PO) antibiotics used after IV treatment for suppression were recorded. Radiographic and clinical presentations of each patient were independently reviewed and graded according to the Cierny-Mader classification system^4^. In addition, the FRI classification, a relatively new classification system, was used to determine if the infection was an active or dormant infection. For FRI cases, the osteomyelitis was classified as confirmed or suspected. FRI confirmed osteomyelitis indicated that the patient had an active infection whereas FRI suspected osteomyelitis indicated that the patient had a history of osteomyelitis but might not have osteomyelitis at presentation. Confirmed FRI was defined by the presence of a draining sinus or 2/5 or more positive cultures obtained at the salvage surgery. Patients with a suspected FRI included those with a history of previous FRI but with no sinus and only 1/5 isolated positive or no positive intraoperative cultures at index salvage surgery.^13^ At latest follow-up, available patients completed a routine follow-up survey. The primary outcome was the ability to control infection without the need for amputation or chronic antibiotic suppression. Failure was defined as need for amputation or chronic antibiotic use due to uncontrolled infection. In the infected nonunion subgroup, the primary outcome was successful bony union where failure was considered the persistence of nonunion.

### Treatment protocol

During the salvage operation, five tissue samples were obtained for culture to confirm the presence of chronic infection. The wound was subsequently debrided down to visibly bleeding bone and soft tissues. Debridement of the infected site was performed with either a single-staged or multi-staged approach based on clinical necessity. It is common in our practice to use external fixation or antibiotic-coated implants to safely perform single-staged surgery. At the surgeon's discretion, local antibiotics were used in the form of antibiotic loaded absorbable beads or antibiotic loaded PMMA coated intramedullary nails. Soft tissue coverage using free tissue transfer was performed by a plastic surgeon if wound closure was deficient. Patients were then given IV, or in rare cases, oral antibiotics with close management by the infectious disease team. The protocol used at our center called for 6 weeks of IV antibiotics. In cases of non-union, the IV antibiotics were followed by oral antibiotics to suppress the infection until bony union was achieved. In certain cases of quinolone sensitive pathogens, six weeks of oral quinolone were used in lieu of IV therapy due to the equivalent bioavailability of these routes of administration. Patients were followed at monthly intervals with physical exam and radiographs, as per routine post-operative protocol, to confirm clinical infection control and bony union. Formal testing such as serum infection markers and contrast MRI were not performed routinely post-surgery to monitor infection.

### Statistical analysis

Continuous variables were reported as means and standard deviations in the descriptive analysis. Frequencies and percentages were used to report descriptive statistics of discrete variables. Demographic and clinical differences were analyzed between patients whose infection was not controlled (“failure”) versus those where the infection was controlled (“non-failure”). A sub-analysis was performed between patients whose initial non-union was healed after salvage treatment versus those whose non-union was not resolved despite salvage surgery. Due to the limited number of patients identified who did not have their infection controlled (n=6), continuous variables were assessed using non-parametric Mann-Whitney U tests. Categorical variables were assessed using Fisher's exact tests. All analyses were performed using two-sided testing with statistical significance was defined as p-values of 0.05 or below. SPSS version 23.0 (IBM Corp., Armonk, NY) was used for the analysis of the study.

## Results

### Study Population

A total of 67 patients (mean age 51.4 years, IQR 17) undergoing limb salvage for treatment of chronic osteomyelitis were identified (Table 2). The majority of the patients had fracture/fusion non-unions of the tibia/ankle with the most common etiology due to trauma (Table 3). Close to 80% of the patients had 2 or more prior surgeries before seeking limb salvage. Active infection was confirmed in 88% of the patients studied. The remaining 12% were treated for suspected infection. The most common organism that grew was MRSE (14 out of 59 patients) (Table 4). On average, 3.6 (s.d. 2.2) salvage surgeries were performed at our center to treat the chronic osteomyelitis. 26 were treated with a single-staged approach while 41 underwent a multi-staged approach. Nearly 70% of the cases did not need soft tissue coverage. Antibiotic beads and antibiotic coated nail were used on occasion, 21% and 15%, respectively. On average, patients were given IV/PO antibiotics for 5.9 weeks (s.d. 0.5). Nonunion patients were given an additional chronic daily antibiotic for an average of 2.7 months (Table 5).

Of the 67 patients that underwent limb salvage treatment, 6 patients did not have their infection controlled. Five patients ultimately needed amputation and one was prescribed chronic antibiotic suppression for life. When compared to the patients with successful limb salvage, a statistically significant number of patients that failed were type II diabetic, neuropathic patients (83%; p < 0.006) (Table 6). Additionally, patients who failed limb salvage had significantly more salvage surgeries compared to patients who had successful limb salvage (5.3 vs 3.4; p = 0.036). All 6 patients who failed limb salvage had confirmed infection. Likewise, 87% of patients that were non-failure had confirmed infection. The use of local antibiotics and duration of IV therapy was not different in the patients that failed.

Of the 54 patients with an infected non-union, 48 had successful healing of their infected non-union. Six patients had persistent non-union. Of the six patients who had persistent non-union, 4 were the same patients who failed to achieve infection control. A statistically significant number of patients who had a persistent non-union were diabetic. Additionally, patients who had persistent non-union had significantly more attempted salvage surgeries compared to patients who healed (5.5 vs 3.5; p = 0.023) (Table 7). Of the patients with persistent non-union after treatment for limb salvage, 50% ultimately underwent amputation and one patient remained on chronic antibiotics. Of the patients that had their non-union healed, 98% of the patients had their infection controlled. One patient healed the bone but had an uncontrolled infection. This patient ultimately had an amputation. A detailed description of all failure patients can be found in Table 8.

A routine post-surgery follow-up questionnaire was available for 72.3% (47/65) of patients, with follow up averaging 3.9 years (range 1.1 to 14.8 years). All but one patient reported they were able to walk. 70% indicated that they ambulated with a limp and 55% reported the need to ambulate with a supportive device. 98% indicated they were satisfied with the treatment and 83% reported they would undergo limb salvage again (Table 9).

## Discussion

Chronic osteomyelitis following lower extremity injuries is a challenging condition to treat where all patients face the possibility of amputation. With the development and improvement of distraction osteogenesis and various grafting techniques, large sections of diseased bone can be removed and reconstructed, infections can be cured, and the affected limb can return the patient to a functional status^14^,^20^. Although positive results have been documented, literature review within the last 30 years has yielded limited published studies with variable results (Table 10). Even fewer studies focused on identifying factors to guide successful limb salvage treatment or help grade potential for success^21^. This study aimed to evaluate the success of our current, multi-disciplinary treatment protocol for osteomyelitis including aggressive debridement, bony stabilization, and 6 weeks of IV (or certain oral) antibiotics (followed by oral suppression until union in cases of non-union).

One of the difficulties in comparing the studies on limb salvage in the treatment of chronic osteomyelitis is the different definitions that authors had set to determine success. Examples include combination of eradication and fracture healing^1^, functional outcome^24^, or absence of amputation^22^. Various classification systems were used to describe the severity of the infection including AO classification, Gustilo classification, and Cierny Mader classification. The distinction between active infection and a history of infection is not documented in many series. The majority of the patients in this series were Cierny Mader Grade 4 (infected non-union), reflecting the complexity of the population that was treated. Active infection was confirmed in 88% (59/67) of the patients studied (54 with culture-positive samples, 5 with draining sinus). The remaining 12% were treated for suspected infection. Limb salvage was considered a success when infection was controlled without the use of chronic antibiotic suppression or amputation. Based on this definition, the effectiveness of our treatment protocol at controlling infection was 91.0%. This is comparable to the previous reported cure rates of 61.9% to 100%^8^,^17^,^22^,^24^. The importance of an integrated team approach has been emphasized in the past. Salvana et al. discussed the roles of nurse practitioners in wound care, infectious disease physicians in antibiotic management and internal medicine physicians in management of chronic pain and depression for ongoing care post-surgery^22^. This multi-disciplinary approach was critical in our treatment protocol as well and included orthopaedic surgeons, plastic surgeons and infectious disease physicians.

While the success in infection control has been documented, limited studies have identified factors associated with outcome. Current recommendations rely primarily on the individual surgeon's experience. Except for Siegel et al, none of the other case series statistically evaluated demographic variables leading to limb salvage failure. Siegel et al found that a history of smoking, age older than 45 years, and multiple and/or intraarticular injuries were potential risk factors leading to poor functional outcomes^24^. Other factors that have been observed to negatively impact the prognosis of chronic osteomyelitis include duration of drainage and extent of debridement^8^. Interestingly, Beals et al found that osteomyelitis involving the distal third of the tibia had a poorer prognosis. The authors suggested that it might be due to the type of microorganisms isolated in their case series (anaerobic bacteria)^1^. However, it has also been recognized that the distal-third tibia has poor fracture healing due to the decreased soft-tissue coverage and naturally diminished vascularity in this region^16^,^23^. Thus, having adequate soft-tissue coverage is an important factor to the success of limb salvage. In our study, the need for soft tissue coverage was not associated with the ability to control infection albeit the numbers were very low. Two factors that did show a statistically significant difference when comparing between the success and failure groups were diabetics with peripheral neuropathy and repeated salvage surgeries. Both carried a higher risk of failure.

The need for more surgeries reflected an effort to aggressively eradicate a difficult infection while the data suggests a lower chance of success with each attempt. It is important to note that the actual number of failure patients in our study was low (n = 6), and a definitive conclusion about when to abandon salvage should not be drawn from these results.

One factor that was suspected to play a role in the overall success of the limb salvage therapy was the grade of the initial infection. However, in this study lacked the power and distribution needed to show that the Cierny Mader grade of osteomyelitis was predictive. In addition, the use of local antibiotics (including antibiotic-loaded beads and antibiotic-loaded, PMMA coated intramedullary nail) was not found to be significant in the success of the limb salvage treatment, again with low power. The efficacy of local antibiotic depots cannot be determined from this investigation.

In addition to treating the infection, limb salvage requires treating bony non-union which often accompanies chronic osteomyelitis^21^. Few studies have documented clearly the initial number of patients presenting with non-unions^1^,^8^, and it is unclear how many of the presenting non-unions healed. In this study, 54 of 67 patients presented with an infected non-union. The ability to heal an infected non-union with our treatment approach was 88.9%. When comparing patients with successful healed non-unions to those with persistent non-union, the results in this case series suggest that the risk factors for failure to unite include number of salvage surgeries, diabetes and uncontrolled infection. The majority of the patients with a healed non-union had a confirmed fracture-related infection (FRI) (90%) and all of those that did not heal had confirmed FRI. This is important to note since it confirms that the majority of patients in both groups were actively infected and needed antibiotic treatment as opposed to the patients with suspected infections that were treated even though they might not have actually needed antibiotic treatment.

While eradication of infection and consolidation of non-union is important, an excellent bone result does not guarantee a similar functional result. The patient's quality of life and ability to participate in society following limb salvage treatment must be considered. Studies have reported between 82.1% to 100% of patients returning to baseline activities following limb salvage^1^,^6^,^24^. However, while a few studies reported greater than 95% ambulation without assistance^12^,^17^ others have been more specific reporting the presence of a residual limp^6^. Chronic pain is another problem facing patients as well^6^,^12^,^22^. Similarly, in the present study, all but one patient confirmed their able to ambulate. However, 70% self-reported that they ambulated with a limp, and 55% reported the need to ambulate with a supportive device suggesting that many still have functional limitations after limb salvage. Despite this, 98% indicated they were satisfied with the treatment and 83% reported they would undergo limb salvage again reasserting their continued rejection of amputation reconstruction. A brief note on the financial burden of limb salvage: Lowenberg et al performed a cost analysis comparing limb salvage and amputation and found that the mean lifetime cost per patient per year after limb salvage was significantly less than the cost for amputation^12^.

One of the notable limitations to this study is the limited number of patients in the failure group (n = 6). Multiple univariate statistical testing could not be performed to evaluate the impact of co-founders. While there were a few factors that had a P-value less than 0.05, the statistical power may actually still be low. As a result, making any definitive comparisons is difficult and conclusions may be premature. However, the fact that there were so few patients in the failure group is an indirect indicator of the success of our limb salvage protocol. In addition, a higher follow-up rate for functional outcomes would have been preferred. Given the importance of functional outcome as an indicator of success with limb salvage technique, a validated lower limb questionnaire should be used in the future for assessment. Despite this, we believe the follow-up surveys were meaningful as they objectively gave the patient's perspective of the surgical capabilities.

## Conclusion

This study demonstrated that limb salvage using an integrated multidisciplinary team approach is a viable and highly successful option in treating complex chronic osteomyelitis and associated non-union involving the lower extremities. Diabetic neuropathy and number of surgical reconstruction attempts are risk factors that may impact the success of a limb salvage undertaking. While infection control and bony union are the primary goals for the treatment team, the functional outcome for the patient is another important measure of success. Further studies addressing this metric will aide in the decision to pursue limb salvage versus amputation.

## Figures and Tables

**Table 1:** Inclusion/Exclusion Criteria

Inclusion Criteria	Exclusion Criteria
Surgical debridement of OM of the tibia, fibula and/or ankle Pre- and Post-operative outcome data Pre- and Post-operative x-rays	Midfoot/forefoot OM Prior amputation Infection involving the knee joint and/or femur

OM = osteomyelitis

**Table 2:** Patient Demographics

Number of Patients	67
Mean Age at Treatment Onset, yr (s.d.)	51.4 (15.4)
**Gender, n (%)**	
Male	40 (60%)
Female	27 (40%)
**Affect Side, n (%)**	
Left	30 (45%)
Right	37 (55%)
**Affected Region, n (%)**	
Ankle only	29 (43%)
Tibia only	25 (37%)
Ankle & Tibia	9 (13%)
Ankle & Hindfoot	4 (6%)
**Smoking, n (%)**	8 (12%)
**Diabetic, n (%)**	13 (19%)
**Neuropathy, n (%)**	19 (28%)
**Vascular Disease, n (%)**	2 (3%)
**Dialysis, n (%)**	1 (1%)
**Pre-existing Bone Disease, n (%)**	3 (4%)
**Other Immuno-compromise, n (%)**	6 (9%)

**Table 3:** Clinical Characteristics

	#
**Etiologies, n (%)**	
Trauma	59 (88%)
Oncologic	3 (4.5%)
Failed Ankle Fusion	2 (3%)
Hematogenous	2 (3%)
Edema/Vascular	1 (1.5%)
**Cierny Osteomyelitis Type, n (%)**	
Type 1	3 (4%)
Type 2	2 (3%)
Type 3	6 (9%)
Type 4 (non-union)	56 (84%)
Hardware Present, n (%)	48 (72%)
Non-union, n (%)	56 (84%)
# of Prior Surgeries, n (%)	
0	1 (1%)
1	13 (19%)
2+	53 (79%)
**Cultured Samples, n (%)**	
Patients with Samples Collected	64 (96%)
Mean # Samples Collected/patient (sd)	5.3 (1.4)
Mean # Positive Samples/patient (sd)	3.9 (2.2)

**Table 4:** Most common microorganisms cultured

Microorganisms	Total #
1. MRSE	14
2. Pseudomonas^	8
3. CoNS	7
4. MSSA 5. Enterococcusº	7 7
6. Enterobacter^+^	6
7. MRSA	6

NOTE: ^Pseudomonas aeruginosa (5), Pseudomonas (unspecified) (2), Pseudomonas fluorescens (1) ºEnterococcus faecalis (4), Enterococcus (unspecified (2), Enterococcus faecium (1) ^+^Enterobacter cloacae (3), Enterobacter amnigenus (1), Enterobacter aerogenes (1), Enterobacter (unspecified) (1)

**Table 5:** Limb Salvage Procedures

	Total (# of Patients w/ Data)	Mean (sd or %)
**# Salvage Surgeries**	67	3.6 (2.2)
**Confirmed Infections**	67	59 (88%)
**Time to Clinical Union (wks)**	30.9	30.9 (16.4)
**Use of Abx Coated IM Nail**	67	10 (15%)
**Use of Abx Beads**	67	14 (21%)
**Soft Tissue Coverage**		
Not needed	67	46 (69%)
At the salvage surgery	67	6 (9%)
Previous to presentation for salvage surgery	67	15 (22%)
**Duration of Acute IV Abx(s) (wks)**	65	5.9 (0.5)
**Duration of PO Abx(s) suppression (mos)**	38	2.7 (2.5)

Confirmed infection = 2 or more positive intra operative cultures. Wks = weeks, Abx = antibiotics, IM = intramedullary, IV = intravenous, mos = months.

**Table 6:** Primary outcome: Success of Infection Control

Parameters	Controlled			Uncontrolled			P-value
	N	Mean	SD	N	Mean	SD	
**Age at Treatment Onset (yr)**	61	51.7	15.7	6	48.8	12.4	0.692
**Sex, n (%)**							
Male	61	37	61%	6	3	50%	0.679
Female	61	24	39%	6	3	50%	
**Cierny Type of OM**							
Type 1	61	3	5%	6	0	0%	0.818
Type 2	61	2	3%	6	0	0%	
Type 3	61	5	8%	6	1	17%	
Type 4	61	51	84%	6	5	83%	
**Affected Region**							
Ankle	61	25	41%	6	4	67%	0.131
Ankle/Tibia	61	7	11%	6	2	33%	
**Diabetics, n (%)**	61	8	13%	6	5	83%	0.001
**Neuropathy, n (%)**	61	14	23%	6	5	83%	0.006
**# Salvage Surgeries**	61	3.4	2.1	6	5.3	2.9	0.036
**Soft Tissue Coverage**							
No	61	41	67%	6	5	83%	0.346
Yes	61	5	8%	6	1	17%	
Previously	61	15	25%	6	0	0%	

**Table 7:** Union v Non-union

Parameters	Final Union (Yes)			Final Union (No)			P-value
	N	Mean	SD	N	Mean	SD	
**Age at Treatment Onset (yr)**	48	50.0	16.1	6	49.2	11.6	0.967
**Sex, n (%)**							
Male	48	30	63%	6	3	50%	0.667
Female	48	18	38%	6	3	50%	
**Cierny Type of OM**							
Type 4	48	48	100%	6	6	100%	
**Etiology**							
Trauma	48	43	90%	6	6	100%	0.984
**Affected Region**							
Ankle	48	22	46%	6	4	67%	0.727
Ankle/Tibia	48	7	15%	6	1	17%	
Tibia	48	17	35%	6	1	17%	
**Diabetics, n (%)**	48	7	15%	6	4	67%	0.012
**Neuropathy, n (%)**	48	13	27%	6	4	67%	0.071
**Confirmed infections**	48	43	90%	6	6	100%	>0.999
**# Salvage Surgeries**	48	3.5	1.7	6	5.5	2.7	0.023
**Infection Controlled?**	48	47	98%	6	2	33%	<0.001
**Amputation?**	48	1	2%	6	3	50%	0.003

**Table 8:** Limb Salvage Failures

	Age	Sex	Affected Side	Affected Part	Diabetic	Neuropathic	Cierny Type	Etiology	Final Union	Reason for Failure
**Infection Control Failures ONLY**										
Patient 1	55	M	L	Ankle	Y	Y	4	Trauma	Yes	Amputation
Patient 2	41	F	L	Ankle/Tibia	Y	Y	3	Trauma	Yes	Amputation
**Bony Union Failures ONLY**										
Patient 3	47	F	L	Ankle	Y	Y	4	Trauma	No	Nonunion
Patient 4	51	M	R	Tibia	N	N	4	Trauma	No	Nonunion
**Infection Control AND Bony Union Failures**										
Patient 5	60	M	L	Ankle/Tibia	Y	Y	4	Trauma	No	Amputation
Patient 6	59	F	L	Ankle	N	N	4	Trauma	No	Amputation
Patient 7	28	F	R	Ankle	Y	Y	4	Trauma	No	Amputation
Patient 8	50	M	R	Ankle	Y	Y	4	Trauma	No	Chronic Infection

**Table 9:** Follow-up Questionnaire

	Total (# of Patients with data)	Mean (%)
Able to Walk, n (%)	47	46 (98%)
Limp, n (%)	47	33 (70%)
Supportive Device, n (%)	47	26 (55%)
Satisfied with Treatment, n (%)	46	45 (98%)
Would Undergo Limb Salvage Again, n (%)	46	38 (83%)

**Table 10:** Literature Review

Authors	Year	No. of patients	Avg Age (yr)	Affected Extremity	Severity of Infection	Success Rate	Avg F/U (yr)	Factors ID for Failure	Functional Outcomes
Esterhai et al	1990	42	35.0	Tibia	Cierny 4 - 42	IC + U - 61.9% Amputations - 4	3.8	Inadequate debridement	N/A
Gayle et al	1992	55	36.0	Tibia	Cierny 3 & 4	IC - 90.9% U - not reported Amputations - 5		N/A	N/A
Patzakis et al	1995	32	40.0	Tibia	Cierny 4 - 32	IC - 100% U - 91% Amputations - 0	2.3	N/A	31/32 ambulate w/o assistance
Dendrinos et al	1995	28	37.4	Tibia	AO Classification	IC - 100% U - 85.7% Amputations - 1	3.3	N/A	18/28 persistent pain 19/28 walk with limp 23/28 returned to work/activities
Siegel et al	2000	46	48.6	Tibia	Cierny 1 - 0 Cierny 2 - 1 Cierny 3 - 9 Cierny 4 - 36	IC - 97.8% U - 95.7% Amputations - 0	5.1	Smoking Advanced age (>45 yo) Multiple and/or intra-articular injuries	Used modified Limb Extremity Outcomes Instruments (AAOS) 43/46 able to perform ADLs as previously described 41/46 reported minimal pain
Beals et al	2005	30	43.6	Tibia	Cierny 1 - 3 Cierny 2 - 5 Cierny 3 - 9 Cierny 4 - 13	IC + U - 90.0% Amputations - 0	6.0	Distal 1/3 of tibia	All returned to pretreatment activity level or better None on chronic pain meds 28/30 ambulate in community
Salvana et al	2005	82	46.5	Various	Cierny 3 & 4	IC - 98.8% U - 92.7% Amputations - 5	4.7	N/A	6/82 on chronic pain meds
Pinzur et al	2012	73	57.9	Foot/Ankle	Not recorded	IC - 93.2% U - not reported Amputations - 4	> 1.0	N/A	N/A
Lowenberg et al	2013	34 (20 - chronic OM)	40.0	Tibia	Not recorded	IC - 100% U - 91.2% Amputations - 0	11.0	N/A	33/34 ambulate w/o assistance 2/34 on chronic pain meds
Halim et al	2016	8	31.5	Tibia	Gustilo Grade III - 8	IC + U - 87.5% Amputations - 1	> 1.0	N/A	N/A

*IC - infection controlled, U - union.
